# Antibiotic susceptibility of *Aggregatibacter actinomycetemcomitans* JP2 in a biofilm

**DOI:** 10.3402/jom.v5i0.20320

**Published:** 2013-05-08

**Authors:** Orit Oettinger-Barak, Stuart G. Dashper, Deanne V. Catmull, Geoffrey G. Adams, Michael N. Sela, Eli E. Machtei, Eric C. Reynolds

**Affiliations:** 1 Melbourne Dental School, Oral Health CRC, Bio21 Institute, University of Melbourne, Parkville, VIC, Australia; 2 Betty and Walter Cohen Chair for Periodontal Research, The Faculty of Dental Medicine, The Hebrew University, Jerusalem, Israel; 3 Department of Periodontology, School of Graduate Dentistry, Rambam Health Care Campus, Haifa, Israel; 4 School of Dental Medicine, Harvard Medical Center, Boston, MA, USA

**Keywords:** Localized aggressive periodontitis, Aggregatibacter actinomycetemcomitans, doxycycline, metronidazole, biofilm

## Abstract

**Background:**

Localized aggressive periodontitis (LAgP) is an inflammatory disease associated with specific bacteria, particularly *Aggregatibacter actinomycetemcomitans*, which can result in early tooth loss. The bacteria grow as a biofilm known as subgingival plaque. Treatment includes mechanical debridement of the biofilm, often associated with empirical antibiotic treatment.

**Objective:**

The aims of this study were to test *in vitro* the sensitivity of *A. actinomycetemcomitans* JP2 during planktonic and biofilm growth to doxycycline and to the combination of metronidazole and amoxicillin, which are two antibiotic protocols commonly used in clinical practice.

**Design:**

Two *in vitro* biofilm models were used to test the effects of the antibiotics: a static 96-well plate assay was used to investigate the effect of these antibiotics on biofilm formation whilst a flow chamber model was used to examine the effect on established biofilms.

**Results:**

Of the antibiotics tested in this model system, doxycycline was most efficacious with a minimal inhibitory concentration (MIC) against planktonic cells of 0.21 mg/L and minimal biofilm inhibitory concentration (MBIC) of 2.10 mg/L. The most commonly prescribed antibiotic regimen, amoxicillin + metronidazole, was much less effective against both planktonic and biofilm cells with an MIC and MBIC of 12.0 mg/L and 20.2 mg/L, respectively. A single treatment of the clinically achievable concentration of 10 mg/L doxycycline to sparse *A. actinomycetemcomitans* biofilms in the flow chamber model resulted in significant decreases in biofilm thickness, biovolume, and cell viability. Dense *A. actinomycetemcomitans* biofilms were significantly more resistant to doxycycline treatment. Low concentrations of antibiotics enhanced biofilm formation.

**Conclusion:**

*A. actinomycetemcomitans* JP2 homotypic biofilms were more susceptible in vitro to doxycycline than amoxicillin + metronidazole.

Localized aggressive periodontitis (LAgP) is a rapidly progressive form of periodontal disease affecting the periodontal tissues of young patients and can result in tooth exfoliation at a young age (1). *Aggregatibacter actinomycetemcomitans* is a Gram-negative capnophilic bacterium that is regarded as a principal pathogen associated with LAgP (1, 2). This bacterium is also associated with systemic infections that can result in endocarditis (3), meningitis, and brain abscesses (4). *A. actinomycetemcomitans* has also been detected in atherotic plaques and in placentas of women suffering from preeclampsia, which is a pregnancy complication evoking high blood pressure and endangering both the mother and the fetus (5).

Although root planing and surgical intervention are the foundations of periodontal therapy, adjunctive antimicrobial chemotherapy can improve the effectiveness of treatment in individuals with difficult to treat types of periodontal diseases, such as aggressive periodontitis (6). Antimicrobial therapy, usually administered systemically, offers several advantages in the treatment of periodontal disease. Not only can it act against pathogens that have invaded soft tissue, but it can also inhibit microorganisms at other sites that are relatively inaccessible, for example, deep, narrow periodontal pockets. The literature describes a number of empirical protocols for the systemic antibiotic treatment of LAgP. These protocols are mostly based on doxycycline (7, 8) or the combination of amoxicillin and metronidazole (9–11). Other protocols involve clindamycin (12), amoxicillin (10, 13), or metronidazole (14, 15) as sole agents. There are currently no universally accepted protocols for the treatment of *A*. *actinomycetemcomitans*-associated periodontitis.

Clinical isolates of *A. actinomycetemcomitans* are known for their ability to form extremely tenacious biofilms *in vitro* (16). The ability to form biofilms is essential for oral bacteria to cause disease, and growth as a biofilm affords many advantages to bacteria. Most importantly in the oral cavity, failure of the bacterium to attach and grow as a biofilm will result in rapid clearance. Biofilms complicate treatment of periodontitis by protecting bacteria from the immune system, decreasing antibiotic/antimicrobial efficacy and allowing the dispersal of planktonic cells to distant sites that can aid reinfection (17). The host inflammatory immune response is not very effective in killing bacteria within biofilms and may result in damage to surrounding tissues due to the chronic release of inflammatory mediators in an effort to overcome the persistent bacterial challenge. The clinical efficacy of antimicrobial agents in the oral cavity is largely dependent upon their ability to penetrate or disrupt biofilms accreted to the tooth surface (dental plaque) and kill bacteria that make up these biofilms. *In vitro* screening of oral antimicrobials is often performed on planktonic bacterial suspensions; however, this is poorly predictive of clinical efficacy (18). More recently, researchers have used biofilm cultures in their *in vitro* testing of oral antimicrobials because of the increased resistance of biofilm bacteria to antimicrobial agents (19–24).

The aims of this *in vitro* study were to evaluate the efficacy of doxycycline versus the combination of amoxicillin with metronidazole against *A. actinomycetemcomitans* JP2 in biofilm models compared with planktonic growth.

## Material and methods

### Bacterial strains and culture conditions

*A. actinomycetemcomitans* strain JP2 (ATCC 700685) was acquired from the American Type Culture Collection. The JP2 strain was originally isolated from subgingival plaque of an aggressive periodontitis patient. *A. actinomycetemcomitans* was cultured on TSBV plates (Tryptic soy-Serum-Bacitracin-Vancomycin) as described by Slots et al. (25) and maintained in an MK3 anaerobic workstation (Don Whitley Scientific, Adelaide, Australia) with gas composition of 5% CO_2_, 5% H_2_, and 90% N_2_ (BOC Gases, Wetherill Park, Australia) at 37°C.

### Susceptibility of planktonic A. actinomycetemcomitans to antibiotics

Exponentially growing *A. actinomycetemcomitans* cells in Brain Heart Infusion (BHI) medium (Becton Dickinson, North Ryde, NSW, Australia) were diluted with fresh medium to give a cell density of 4×10^8^ cells/mL. Doxycycline and amoxicillin were dissolved in de-ionized water whilst metronidazole was dissolved in 5% dimethylsulfoxide in de-ionized water and mixed with the dissolved amoxicillin in a 1:1 ratio. The bacterial suspension (20 µL) plus 20 µL of each dissolved antibiotic were added to 160 µL of BHI medium in 96-well flat bottom plates, giving final antibiotic concentrations ranging between 0.01–1000 mg/L. The plates were incubated at 37°C with periodic shaking and growth was monitored for 24 h by measuring Absorbance at a wavelength of 620 nm (AU_620_) using an iEMS microplate reader (Labsystems OY Research Technologies Division, Helsinki, Finland). The minimal inhibitory concentration (MIC) was calculated by linear regression of the AU_620_ versus antibiotic concentration data. Fiducial limits were estimated by converting the 95% prediction intervals for the linear regression at AU_620_=0 (26). All antibiotics were obtained from Sigma-Aldrich Corporation (St. Louis, MO, USA).

### Effects of antibiotics on A. actinomycetemcomitans biofilm formation

To determine the effect of antibiotics on biofilm formation a static 96-well plate assay was used that was adapted from Izano et al. (23) using the same growth medium, inoculation protocol, and antibiotic solutions as described above. The plates were incubated for 24 h at 37°C after which the adherent biofilms were rinsed twice with 210 µL of de-ionized water to remove loosely attached cells, followed by 5 min incubation with 0.1% crystal violet. The crystal violet stained biofilm was then dissolved in 99% ethanol for 20 min, through repeated pipetting before transfer to a new 96-well plate. Quantification of the biofilms was carried out by measuring AU_620_. The minimal biofilm inhibitory concentrations (MBIC) were calculated by linear regression of the AU_620_ versus antibiotic concentration data. Fiducial limits were estimated by converting the 95% prediction intervals for the linear regression at AU_620_=0 (21). The AU_620_ values at different dose levels were compared by one-way analysis of variance (ANOVA). When the overall *F*-test was significant, the Dunnett post-hoc test was used to compare whether the mean U_620_ values at different concentrations were significantly greater than the mean U_620_ values for the untreated control. The biofilms were also analyzed for viable cells by culture analysis after serial dilution using TSBV agar as described previously (24).

### Flow cell biofilm culture and confocal laser scanning microscopy analysis

Based on the results of the static biofilm assay the most effective antibiotic was selected and used in a flow chamber model, to determine the effects of the antibiotic on sparse immature and more established *A. actinomycetemcomitans* biofilms.

#### Sparse biofilm model

The biofilm culture of *A. actinomycetemcomitans* JP2 in a three-channel flow cell system (Stovall Life Science, Greensboro, NC, USA) located in an MK3 workstation was based on that described by Dashper et al. (24). The system was inoculated with 1 mL of an exponentially growing *A. actinomycetemcomitans* culture at 4×10^8^ cells/mL diluted 1:10 with fresh BHI. The system was incubated for 1 h prior to constant flow (0.2 mL/min) of 35% strength BHI which continued for 24 h. To determine the effect of the antibiotic on these *A. actinomycetemcomitans* JP2 biofilms, 1 mL of 10 mg/L doxycycline dissolved in sterile water, or sterile water as a control, was injected into each channel of the system and incubated for 30 min. The flow of medium was then resumed for another 10 min to wash off any unbound cells, and the adherent biofilms were stained with BacLight stain (Molecular Probes, Invitrogen Corporation, Carlsbad, CA, USA) *in situ* as described previously (22).

#### Dense biofilm model

For the dense biofilm model, the three-channel flow cell methodology described above was used, except that a 1:50 diluted *A. actinomycetemcomitans* inoculum and 50% strength BHI medium were introduced. Confocal laser scanning microscopy Confocal laser scanning microscopy (CLSM) of the bacterial biofilms was carried out on an LSM Meta 510 Confocal Microscope with an inverted stage (Carl-Zeiss, Oberkochen, Germany) as described previously (22). All CLSM images were analyzed using COMSTAT software (27), and the biometric data were statistically analyzed using the unpaired Student's *t*-test.

## Results

### Susceptibility of A. actinomycetemcomitans to antibiotics

Doxycycline and the combination of amoxicillin with metronidazole were evaluated to determine the MIC and MBIC. Of these two antibiotic treatments, doxycycline was the most efficacious with an MIC against planktonic cells of 0.21 mg/L and an MBIC of 2.10 mg/L, while the combination of metronidazole and amoxicillin was much less effective against planktonic cells and biofilms (Table 1). In the static 96-well plate model after 24 h of incubation, *A. actinomycetemcomitans* formed biofilms that comprised 3.79±0.22×10^6^ cells per well as determined by cultural analysis. Interestingly, at low concentrations both doxycycline and the combination of amoxicillin and metronidazole significantly enhanced biofilm formation. At levels of up to 1.0 mg/L, doxycycline significantly enhanced biofilm formation when compared with the untreated control (Table 2). There were statistically significant differences in the mean AU_620_ values of the biofilms between concentrations (ANOVA *F*-test, *p*<0.001), with the mean AU_620_ values increasing from 0.34±0.08 for the untreated control to 0.79±0.19 at 0.05 mg/L (*p* = 0.002) and 0.83±0.19 at 1.0 mg/L (*p* = 0.001). Amoxicillin+metronidazole treatment resulted in an even more significant enhancement of *A. actinomycetemcomitans* biofilm formation (Table 2) at concentrations up to 10 mg/L (*p*<0.001). The mean AU_620_ values of the biofilms increased from 0.39±0.03 for the untreated control to 0.81±0.22 at 1.0 mg/L (*p* = 0.01), 0.76±0.08 at 5.0 mg/L (*p* = 0.02), and 1.65±0.19 at 10.0 mg/L (*p*<0.001).

**Table 1:** The minimal inhibitory concentration (MIC) and minimal biofilm inhibitory concentration (MBIC) of doxycycline and amoxicillin + metronidazole determined using 96-well plate static assays

Antibiotic	MIC^a^ (mg/L)	MBIC (mg/L)
Doxycycline	0.21 (0.19–0.23)	2.10 (1.60–2.80)
Amoxicillin+metronidazole (1:1)	12.0 (10.5–13.5)	20.2 (17.7–23.2)

a MIC and MBIC were determined by linear regression using a minimum of three biological replicates. The fiducial limits shown in parentheses were determined by converting the 95% prediction intervals for the linear regression at AU_620_=0.

**Table 2:** Effect of low antibiotic concentrations on biofilm formation using 96-well plate static assays

Doxycycline concentration (mg/L)	0	0.05	1.0	
Biofilm formation (AU_620_)	0.34±0.08	0.79±0.19*	0.83±0.19**	
Amoxicillin + metronidazole concentration (mg/L)	0	1.0	5.0	10
Biofilm formation (AU_620_) values	0.39±0.03	0.81±0.22**	0.76±0.08**	1.65±0.19**

* ANOVA *F*-test for the difference in reading relative to the 0 concentration, *p*<0.002.

** ANOVA *F*-test for the difference in reading relative to the 0 concentration, *p*≤0.001.

Based on the MIC and MBIC results, doxycycline was selected to determine its effect on *A. actinomycetemcomitans* biofilms in the flow cell model. To more closely mimic the clinical environment, a doxycycline concentration of 10 mg/L was chosen, as this concentration has been shown to be achievable in gingival crevicular fluid (GCF) (28).

### Flow cell biofilm culture and CLSM analysis

#### Sparse biofilm

When *A. actinomycetemcomitans* was cultured in the flow cell system for 24 h with 35% BHI it formed relatively sparse biofilms with a maximum thickness of 12.7 µm (Table 3). These biofilms contained a high percentage of viable cells as demonstrated via Live/Dead staining (red cells represent cells with disrupted membranes ‘dead’ and green cells those with intact cell membranes ‘live’). A single 30 min treatment of these *A. actinomycetemcomitans* biofilms with 10 mg/L doxycycline resulted in a significant 24% reduction in maximum biofilm thickness and a 59% reduction in average biofilm thickness (Table 3). Statistically significant reductions in biovolume (51%) and viability (24%) following doxycycline administration were determined (Table 3). Surface area to biovolume ratio did not change and the roughness coefficient Ra increased subsequent to doxycycline administration (*p*<0.05) (Table 3).

**Table 3:** Effect of a single treatment of 10 mg/L doxycycline on established *A. actinomycetemcomitans* biofilms in a three-channel flow cell system. Dense biofilms were produced using inocula of 8 x 10^6^ cells in 50% BHI and sparse biofilms were produced using inocula of 4 x 10^7^ in 35% BHI. The biometric parameters were obtained using COMSTAT analysis of CLSM images and are presented as means±standard deviations of three biological replicates

	Sparse biofilms		Dense biofilms	
		
Biofilm parameter	Control	Doxycycline 10 mg/L	Control	Doxycycline 10 mg/L
Biovolume (µm^3^/µm^2^)	0.30± 0.07	0.147±0.08^a^ (−51%)	25.64±3.89	18.96±4.56 (−26%)
Average thickness of biofilm (µm)	0.34±0.09	0.14±0.09^a^ (−59%)	31.67±3.36	22.82±3.78^a^ (−28%)
Maximum thickness of biofilm (µm)	12.67±0.23	9.60±1.83^a^ (−24%)	40.77±6.49	34.67±6.11 (−15%)
Surface area: biovolume ratio (µm^2^/µm^3^)	4.05±0.61	4.45±0.44	3.15±1.6	2.71±0.56
Roughness coefficient Ra (unitless)	1.88±0.04	1.94±0.03^a^	0.15±0.04	0.20±0.04
% Viability	81.0±4.6	61.5±7.5^a^ (−24%)	89.1±3.3	78.4±9.2 (−12%)

a Significantly different (*p*<0.05) compared with the relevant control using unpaired Student's *t*-test.

#### Dense biofilm

**Fig. 1:**
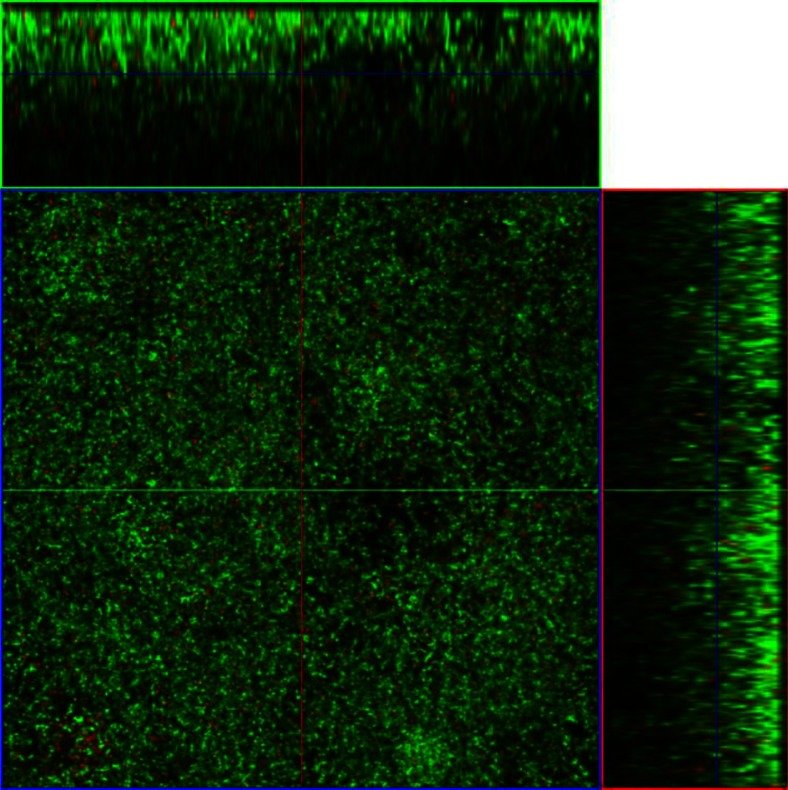
CLSM images of a representative section of an *A. actinomycetemcomitans* 24 h dense biofilm grown in a flow cell and stained with BacLight stain. Horizontal (x–y) optodigital sections, each 2 µm thick over the entire thickness of the biofilm (z), were imaged using a 63× objective at 512 by 512 pixels (0.28 µm per pixel), with each frame at 143.86 µm (x) by 143.86 µm (y). Live cells fluoresce green; dead cells fluoresce red.

**Fig. 2:**
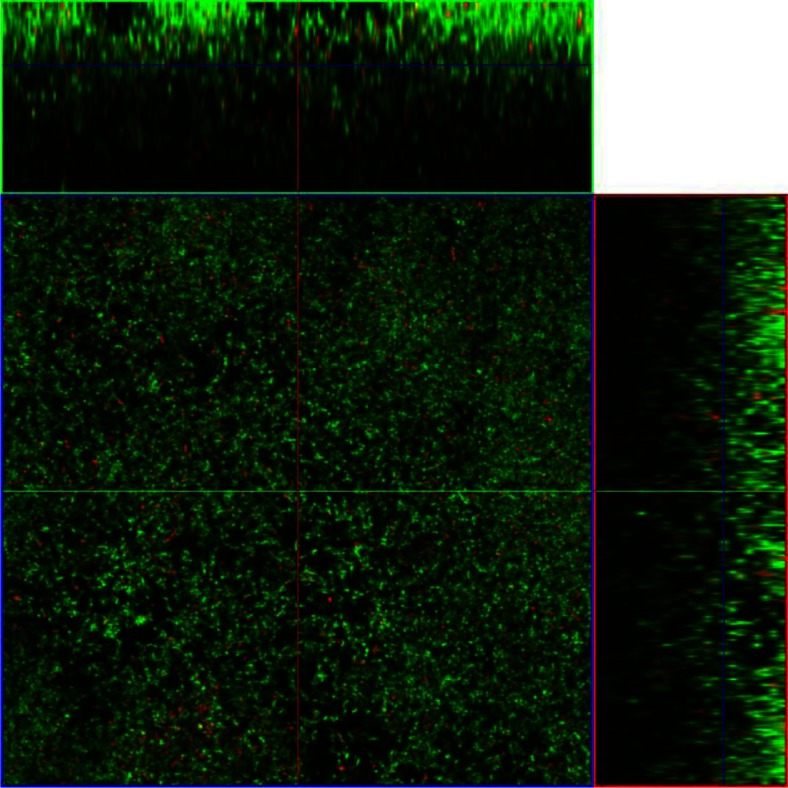
CLSM images of a representative section of an *A. actinomycetemcomitans* 24 h dense biofilm cultured in a flow cell then treated with 10 mg/L doxycycline for 30 min. The biofilm was stained with BacLight stain. Horizontal (x–y) optodigital sections, each 2 µm thick over the entire thickness of the biofilm (z), were imaged using a 63× objective at 512 by 512 pixels (0.28 µm per pixel), with each frame at 143.86 µm (x) by 143.86 µm (y). Live cells fluoresce green; dead cells fluoresce red.

When cultured for 24 h in the flow cell system with 50% BHI *A. actinomycetemcomitans* produced dense, structured biofilms with a maximum thickness of 40.8 µm (Table 3, Fig. 1). Addition of 10 mg/L doxycycline to these mature *A. actinomycetemcomitans* biofilms resulted in a significant 28% reduction in average biofilm thickness (Table 3, Fig. 2). All other biometric parameters analyzed did not show any significant effect of doxycycline treatment compared with the untreated biofilms despite a 12% decrease in viability and 26% reduction in biovolume (Table 3). There were no significant changes in surface area to biovolume ratio and the roughness coefficient Ra subsequent to doxycycline administration (Table 3).

## Discussion

In this *in vitro* study two commonly prescribed antibiotic protocols were tested for their effectiveness against *A. actinomycetemcomitans* JP2 in static 96-well planktonic and biofilm assays. It has been proposed that *A. actinomycetemcomitans* can be grouped into three major phylogenetic lineages: serotype b strains, serotype c strains, and serotype a, d, e, and f strains. The distribution of *A. actinomycetemcomitans* serotypes appears to be related to geographical location and ethnicity (29, 30). Different serotypes have differing virulence, with some studies suggesting that the JP2 clone of serotype b strain has increased virulence. This clone is characterized by a 530-bp deletion from the promoter region of the leukotoxin gene operon, which gives rise to considerably enhanced leukotoxin activity (31). The JP2 clone is attributed to descendants of North African populations, but is also prevalent in LAgP patients from other geographical locations (32, 33). It has been shown that *A. actinomycetemcomitans* JP2 is associated with initiation of periodontal tissue attachment loss (34), and eradication of JP2 provides better outcomes of periodontal treatment (35–37).

Of the antibiotics examined in this study, doxycycline produced the best results, inhibiting both planktonic and biofilm growth at relatively low concentrations (Table 1). Eick et al. (38) reported the doxycycline MIC for planktonic growth of *A. actinomycetemcomitans* as 2 mg/L and the MBIC after 48 h of biofilm culture as 20 mg/L. Whilst Pajukanta et al. (39) reported doxycycline MICs in the range of 0.25 to 2.0 mg/L using an agar plate based assay against a variety of clinical strains. The Pajukanta et al. (39) data are consistent with the MBIC of 2.1 mg/L against static 24 h biofilms obtained in this study, which is a clinically achievable concentration. Doxycycline concentrations in GCF have been shown to reach up to 10 µg/mL following a 200 mg per oral dose (28), but usually lower levels of 2.4–2.5 µg/mL have been reported (40, 41). However, utilizing controlled release load-delivery devices containing doxycycline, levels in GCF can exceed 1 mg/mL (40). GCF antibiotic concentrations represent the maximum achievable concentrations in the periodontal pocket, which is the target of antibiotic activity. The efficacious usage of doxycycline in conjunction with periodontal treatment has been previously described, and it has recently been reported as a successful treatment regimen for young Israeli LAgP patients (42). Several studies have demonstrated a suppression of the pathogenic subgingival microbiota during and after doxycycline/tetracycline administration associated with clinical improvement (43–45). However, many others have shown that the antibiotics failed to completely eliminate periodontal pathogens from periodontal pockets (46–49). One of the causes for treatment failure may be the emergence of resistant human pathogens as a result of the widespread use of antibiotics (50, 51). An increase in resistance of *A. actinomycetemcomitans* to tetracycline has been reported (52). As emergence of resistant strains may have occurred after the type strain ATCC 700685 was isolated, the results of this study should be confirmed with recent clinical isolates. Furthermore, clinical studies also should be conducted to confirm the efficacy of therapy with these antibiotics.

Unfortunately very few clinical studies have been conducted using doxycycline despite its widespread clinical use. In the present study, the combination of amoxicillin and metronidazole was also analyzed as it is a commonly used treatment for periodontitis cases involving *A. actinomycetemcomitans* (9, 53), and has been shown to be efficacious in the treatment of *A. actinomycetemcomitans-*associated periodontal disease (53, 54). A synergistic interaction in which amoxicillin enhances metronidazole uptake by *A. actinomycetemcomitans* has been reported (55); however, there are concerns that *A. actinomycetemcomitans* strains are developing resistance to both metronidazole and amoxicillin (53, 56). The MIC of 12.0 mg/L obtained in this study for a 1:1 ratio of amoxicillin+metronidazole is consistent with previously reported MIC values for metronidazole of 2.0–64 mg/L (39) and 10–40 mg/L (55). The MBIC of a 1:1 ratio of amoxicillin+metronidazole of over 20 mg/L that we obtained in this study is unlikely to be achievable clinically and as such was not further tested.

To produce *in vitro* data that are more predictive of clinical efficacy against established biofilms it is important to incorporate hydrodynamic forces which are continuously present in the oral cavity (57). These forces can exert important shear and clearance effects (58). The flow cell model with CLSM imaging described in this study allows a short exposure of biofilms to the antimicrobial agent and has been used previously to study the effect of a novel antimicrobial agent on *Porphyromonas gingivalis*. *A*. *actinomycetemcomitans* formed structured biofilms in these flow cells and the biovolume of the biofilm after 24 hours of incubation was dependent on dilution of the growth medium (Table 3). The biofilms formed in this study were monospecific so as best to model the direct effect of the antibiotics on the target species, *A*. *actinomycetemcomitans*. *In vivo* the presence of other species of bacteria in the biofilm may have confounding effects on antibiotic susceptibility, and this needs to be considered when relating these data to clinical application. Application of a single dose of doxycycline to sparse *A*. *actinomycetemcomitans* biofilms resembling early stage biofilm development in the oral cavity was effective causing a significant >50% reduction in average biofilm thickness and biovolume as well as a 24% decrease in viability. It is likely that any decrease in viability will be underestimated in our analysis as the Live/Dead stain is reliant on membrane damage to predict loss of viability and as doxycycline works by inhibiting protein synthesis, membrane integrity may not be compromised in the relatively short period of these assays. Doxycycline treatment of denser established biofilms was far less effective leading only to a significant reduction in average biofilm thickness. These data are consistent with those of Takahashi et al. (59) who showed reduced susceptibility of *A*. *actinomycetemcomitans* to antibiotics with maturation of the biofilm. As sparse biofilms develop just after mechanical debridement and mature into denser biofilms if sufficient nutrients are available, it is important to emphasize that while antibiotic administration might be beneficial in assisting in the suppression of *A*. *actinomycetemcomitans* in the periodontal pocket, effective mechanical removal of biofilms as part of the periodontal treatment is crucial prior to chemotherapy.

An interesting and novel phenomenon observed for both antibiotic protocols tested in this study was enhancement of *A. actinomycetemcomitans* biofilm formation at the lower antibiotic concentrations. An increase in biomass has previously been described for newly formed *Staphylococcus epidermidis* biofilms in the presence of vancomycin (60). Those authors suggested that this was due to the thickening of the staphylococcal cell wall in response to the antibiotic. An alternative explanation is that exposure to low concentrations of vancomycin affected the expression of genes involved in biofilm production. In support of this hypothesis, sub-inhibitory concentrations of tetracycline upregulated the expression of the *icaADBC* operon leading to the synthesis of a polysaccharide intercellular adhesin and biofilm formation by *S. epidermidis* (61). Therefore, it is possible that the antibiotics tested in the current study have a similar effect on expression of *A. actinomycetemcomitans* genes that are involved in biofilm formation.

In summary, doxycycline proved to be highly effective at inhibiting *A. actinomycetemcomitans* planktonic growth and monospecies biofilm formation *in vitro* and had significant effects against relatively sparse established biofilms. Low concentrations of antibiotics enhanced *A. actinomycetemcomitans* biofilm formation.

## References

[1] Armitage GC (1999). Development of a classification system for periodontal diseases and conditions. Ann Periodontol.

[2] Faveri M, Figueiredo LC, Duarte PM, Mestnik MJ, Mayer MP, Feres M (2009). Microbiological profile of untreated subjects with localized aggressive periodontitis. J Clin Periodontol.

[3] Wang CY, Wang HC, Li JM, Wang JY, Yang KC, Ho YK (2010). Invasive infections of *Aggregatibacter (Actinobacillus) actinomycetemcomitans*. J Microbiol Immunol Infect.

[4] Stepanovic S, Tosic T, Savic B, Jovanovic M, K'Ouas G, Carlier JP (2005). Brain abscess due to *Actinobacillus actinomycetemcomitans*. APMIS.

[5] Barak S, Oettinger-Barak O, Machtei EE, Sprecher H, Ohel G (2007). Evidence of periopathogenic microorganisms in placentas of women with preeclampsia. J Periodontol.

[6] Slots J (2004). Systemic antibiotics in periodontics. J Periodontol.

[7] Mandell RL, Tripodi LS, Savitt E, Goodson JM, Socransky SS (1986). The effect of treatment on *Actinobacillus actinomycetemcomitans* in localized juvenile periodontitis. J Periodontol.

[8] Akincibay H, Orsal SO, Sengun D, Tozum TF (2008). Systemic administration of doxycycline versus metronidazole plus amoxicillin in the treatment of localized aggressive periodontitis: a clinical and microbiologic study. Quintessence Int.

[9] van Winkelhoff AJ, Rodenburg JP, Goene RJ, Abbas F, Winkel EG, de Graaff J (1989). Metronidazole plus amoxycillin in the treatment of *Actinobacillus actinomycetemcomitans* associated periodontitis. J Clin Periodontol.

[10] Rooney J, Wade WG, Sprague SV, Newcombe RG, Addy M (2002). Adjunctive effects to non-surgical periodontal therapy of systemic metronidazole and amoxycillin alone and combined. A placebo controlled study. J Clin Periodontol.

[11] Winkel EG, Van Winkelhoff AJ, Timmerman MF, Van der Velden U, Van der Weijden GA (2001). Amoxicillin plus metronidazole in the treatment of adult periodontitis patients. A double-blind placebo-controlled study. J Clin Periodontol.

[12] Sigusch B, Beier M, Klinger G, Pfister W, Glockmann E (2001). A 2-step non-surgical procedure and systemic antibiotics in the treatment of rapidly progressive periodontitis. J Periodontol.

[13] Kunihira DM, Caine FA, Palcanis KG, Best AM, Ranney RR (1985). A clinical trial of phenoxymethyl penicillin for adjunctive treatment of juvenile periodontitis. J Periodontol.

[14] Saxen L, Asikainen S (1993). Metronidazole in the treatment of localized juvenile periodontitis. J Clin Periodontol.

[15] Noyan U, Yilmaz S, Kuru B, Kadir T, Acar O, Buget E (1997). A clinical and microbiological evaluation of systemic and local metronidazole delivery in adult periodontitis patients. J Clin Periodontol.

[16] Fine DH, Furgang D, Kaplan J, Charlesworth J, Figurski DH (1999). Tenacious adhesion of *Actinobacillus actinomycetemcomitans* strain CU1000 to salivary-coated hydroxyapatite. Arch Oral Biol.

[17] Cvitkovitch DG, Li YH, Ellen RP (2003). Quorum sensing and biofilm formation in Streptococcal infections. J Clin Invest.

[18] Larsen T (2002). Susceptibility of *Porphyromonas gingivalis* in biofilms to amoxicillin, doxycycline and metronidazole. Oral Microbiol Immunol.

[19] Costerton JW, Stewart PS, Greenberg EP (1999). Bacterial biofilms: a common cause of persistent infections. Science.

[20] Fine DH, Furgang D, Barnett ML (2001). Comparative antimicrobial activities of antiseptic mouthrinses against isogenic planktonic and biofilm forms of *Actinobacillus actinomycetemcomitans*. J Clin Periodontol.

[21] Shapiro S, Giertsen E, Guggenheim B (2002). An *in* vitro oral biofilm model for comparing the efficacy of antimicrobial mouthrinses. Caries Res.

[22] Dashper S, Ang CS, Liu SW, Paolini R, Veith P, Reynolds E (2010). Inhibition of *Porphyromonas gingivalis* biofilm by oxantel. Antimicrob Agents Chemother.

[23] Izano EA, Wang H, Ragunath C, Ramasubbu N, Kaplan JB (2007). Detachment and killing of *Aggregatibacter actinomycetemcomitans* biofilms by dispersin B and SDS. J Dent Res.

[24] Takayama S, Saitoh E, Kimizuka R, Yamada S, Kato T (2009). Effect of eel galectin AJL-1 on periodontopathic bacterial biofilm formation and their lipopolysaccharide-mediated inflammatory cytokine induction. Int J Antimicrob Agents.

[25] Slots J (1982). Selective medium for isolation of *Actinobacillus actinomycetemcomitans*. J Clin Microbiol.

[26] Draper NR, Smith H (1981). Applied regression analysis.

[27] Heydorn A, Nielsen AT, Hentzer M, Sternberg C, Givskov M, Ersboll BK (2000). Quantification of biofilm structures by the novel computer program COMSTAT. Microbiology.

[28] Pascale D, Gordon J, Lamster I, Mann P, Seiger M, Arndt W (1986). Concentration of doxycycline in human gingival fluid. J Clin Periodontol.

[29] Kim TS, Frank P, Eickholz P, Eick S, Kim CK (2009). Serotypes of *Aggregatibacter actinomycetemcomitans* in patients with different ethnic backgrounds. J Periodontol.

[30] Tinoco EM, Sivakumar M, Preus HR (1998). The distribution and transmission of *Actinobacillus actinomycetemcomitans* in families with localized juvenile periodontitis. J Clin Periodontol.

[31] Brogan JM, Lally ET, Poulsen K, Kilian M, Demuth DR (1994). Regulation of *Actinobacillus actinomycetemcomitans* leukotoxin expression: analysis of the promoter regions of leukotoxic and minimally leukotoxic strains. Infect Immun.

[32] Haubek D, Dirienzo JM, Tinoco EM, Westergaard J, Lopez NJ, Chung CP (1997). Racial tropism of a highly toxic clone of *Actinobacillus actinomycetemcomitans* associated with juvenile periodontitis. J Clin Microbiol.

[33] Haubek D, Poulsen K, Kilian M (2007). Microevolution and patterns of dissemination of the JP2 clone of *Aggregatibacter (Actinobacillus) actinomycetemcomitans*. Infect Immun.

[34] Haubek D, Ennibi OK, Poulsen K, Vaeth M, Poulsen S, Kilian M (2008). Risk of aggressive periodontitis in adolescent carriers of the JP2 clone of *Aggregatibacter (Actinobacillus) actinomycetemcomitans* in Morocco: a prospective longitudinal cohort study. Lancet.

[35] Cortelli SC, Costa FO, Kawai T, Aquino DR, Franco GC, Ohara K (2009). Diminished treatment response of periodontally diseased patients infected with the JP2 clone of *Aggregatibacter (Actinobacillus) actinomycetemcomitans*. J Clin Microbiol.

[36] Rylev M, Bek-Thomsen M, Reinholdt J, Ennibi OK, Kilian M (2011). Microbiological and immunological characteristics of young Moroccan patients with aggressive periodontitis with and without detectable *Aggregatibacter actinomycetemcomitans* JP2 infection. Mol Oral Microbiol.

[37] Rylev M, Kilian M (2008). Prevalence and distribution of principal periodontal pathogens worldwide. J Clin Periodontol.

[38] Eick S, Pfister W (2004). Efficacy of antibiotics against periodontopathogenic bacteria within epithelial cells: an *in vitro* study. J Periodontol.

[39] Pajukanta R, Asikainen S, Saarela M, Alaluusua S, Jousimies-Somer H (1993). *In vitro* antimicrobial susceptibility of different serotypes of *Actinobacillus actinomycetemcomitans*. Scand J Dent Res.

[40] Stoller NH, Johnson LR, Trapnell S, Harrold CQ, Garrett S (1998). The pharmacokinetic profile of a biodegradable controlled-release delivery system containing doxycycline compared to systemically delivered doxycycline in gingival crevicular fluid, saliva, and serum. J Periodontol.

[41] Lavda M, Clausnitzer CE, Walters JD (2004). Distribution of systemic ciprofloxacin and doxycycline to gingiva and gingival crevicular fluid. J Periodontol.

[42] Machtei EE, Younis MN (2008). The use of 2 antibiotic regimens in aggressive periodontitis: comparison of changes in clinical parameters and gingival crevicular fluid biomarkers. Quintessence Int.

[43] Christersson LA, Zambon JJ (1993). Suppression of subgingival *Actinobacillus actinomycetemcomitans* in localized juvenile periodontitis by systemic tetracycline. J Clin Periodontol.

[44] Haffajee AD, Dibart S, Kent RL, Socransky SS (1995). Clinical and microbiological changes associated with the use of 4 adjunctive systemically administered agents in the treatment of periodontal infections. J Clin Periodontol.

[45] Walker CB, Godowski KC, Borden L, Lennon J, Nango S, Stone C (2000). The effects of sustained release doxycycline on the anaerobic flora and antibiotic-resistant patterns in subgingival plaque and saliva. J Periodontol.

[46] Asikainen S, Jousimies-Somer H, Kanervo A, Saxen L (1990). The immediate efficacy of adjunctive doxycycline in treatment of localized juvenile periodontitis. Arch Oral Biol.

[47] Mombelli A, Tonetti M, Lehmann B, Lang NP (1996). Topographic distribution of black-pigmenting anaerobes before and after periodontal treatment by local delivery of tetracycline. J Clin Periodontol.

[48] Feres M, Haffajee AD, Goncalves C, Allard KA, Som S, Smith C (1999). Systemic doxycycline administration in the treatment of periodontal infections (I). Effect on the subgingival microbiota. J Clin Periodontol.

[49] Wong MY, Lu CL, Liu CM, Hou LT (1999). Microbiological response of localized sites with recurrent periodontitis in maintenance patients treated with tetracycline fibers. J Periodontol.

[50] Walker CB (1996). The acquisition of antibiotic resistance in the periodontal microflora. Periodontol 2000.

[51] van Winkelhoff AJ, Winkel EG, Barendregt D, Dellemijn-Kippuw N, Stijne A, van der Velden U (1997). beta-Lactamase producing bacteria in adult periodontitis. J Clin Periodontol.

[52] Rodrigues RM, Goncalves C, Souto R, Feres-Filho EJ, Uzeda M, Colombo AP (2004). Antibiotic resistance profile of the subgingival microbiota following systemic or local tetracycline therapy. J Clin Periodontol.

[53] van Winkelhoff AJ, Tijhof CJ, de Graaff J (1992). Microbiological and clinical results of metronidazole plus amoxicillin therapy in *Actinobacillus actinomycetemcomitans*-associated periodontitis. J Periodontol.

[54] Walker C, Karpinia K (2002). Rationale for use of antibiotics in periodontics. J Periodontol.

[55] Pavicic MJ, van Winkelhoff AJ, Pavicic-Temming YA, de Graaff J (1994). Amoxycillin causes an enhanced uptake of metronidazole in Actinobacillus actinomycetemcomitans: a mechanism of synergy. J Antimicrob Chemother.

[56] Ardila CM, Lopez MA, Guzman IC (2010). High resistance against clindamycin, metronidazole and amoxicillin in Porphyromonas gingivalis and Aggregatibacter actinomycetemcomitans isolates of periodontal disease. Med Oral Patol Oral Cir Bucal.

[57] Larsen T, Fiehn NE (1995). Development of a flow method for susceptibility testing of oral biofilms *in vitro*. APMIS.

[58] Cummins D, Creeth JE (1992). Delivery of antiplaque agents from dentifrices, gels, and mouthwashes. J Dent Res.

[59] Takahashi N, Ishihara K, Kato T, Okuda K (2007). Susceptibility of *Actinobacillus actinomycetemcomitans* to six antibiotics decreases as biofilm matures. J Antimicrob Chemother.

[60] Cargill JS, Upton M (2009). Low concentrations of vancomycin stimulate biofilm formation in some clinical isolates of *Staphylococcus epidermidis*. J Clin Pathol.

[61] Rachid S, Ohlsen K, Witte W, Hacker J, Ziebuhr W (2000). Effect of subinhibitory antibiotic concentrations on polysaccharide intercellular adhesin expression in biofilm-forming *Staphylococcus epidermidis*. Antimicrob Agents Chemother.

